# *Notes from the Field:* Overdose Deaths with Carfentanil and Other Fentanyl Analogs Detected — 10 States, July 2016–June 2017

**DOI:** 10.15585/mmwr.mm6727a4

**Published:** 2018-07-13

**Authors:** Julie O’Donnell, R. Matthew Gladden, Christine L. Mattson, Mbabazi Kariisa

**Affiliations:** 1 Division of Unintentional Injury Prevention, National Center for Injury Prevention and Control, CDC.

Fentanyl and fentanyl analogs are increasingly involved in opioid overdose deaths, and new fentanyl analogs continue to be identified (*1*). Carfentanil, the most potent fentanyl analog detected in the United States, is intended for sedation of large animals and is estimated to have 10,000 times the potency of morphine (*2*). It has recently been reported in an alarming number of deaths in some states. Ohio reported nearly 400 carfentanil-involved deaths during July–December 2016, and Florida reported >500 such deaths for all of 2016 (*3*,*4*). 

CDC funds 32 states and the District of Columbia (DC) to abstract detailed data on opioid overdose deaths from death certificates and medical examiner and coroner reports through the State Unintentional Drug Overdose Reporting System (SUDORS). Twelve states began reporting in August 2017, and 20 states and DC will begin reporting in August 2018.* CDC analyzed trends in overdose deaths testing positive for carfentanil and other fentanyl analogs during July 2016–June 2017 in 10 SUDORS states (Kentucky, Maine, Massachusetts, New Hampshire, New Mexico, Ohio, Oklahoma, Rhode Island, West Virginia, and Wisconsin).^†^ States abstract data on all substances (both opioids and nonopioids) that contributed to death, as well as all substances for which the decedent tested positive.^§^

During July 2016–June 2017, among 11,045 opioid overdose deaths, 2,275 (20.6%) decedents tested positive for any fentanyl analog, and 1,236 (11.2%) tested positive for carfentanil. Fourteen different fentanyl analogs were detected.^¶^ Among overdose deaths with fentanyl analogs detected, the analogs were determined by medical examiners or coroners to have contributed to the death in >95% of deaths. During the first half of 2017, the number of deaths with any fentanyl analog detected (1,511) nearly doubled, compared with the number during the second half of 2016 (764); deaths with carfentanil detected increased 94%, from 421 to 815. The proportions of deaths with any fentanyl analog or with carfentanil detected nearly doubled during this period.

Ohio reported the largest numbers and most substantial increases in deaths with any fentanyl analog detected, including carfentanil (Figure). The number of carfentanil deaths in Ohio initially peaked in September 2016 (86 deaths), decreased during October 2016–February 2017, and peaked again in April 2017 (218 deaths). Changes in the number of deaths with any fentanyl analog detected mirrored changes in deaths with carfentanil detected, except during October 2016–February 2017, when deaths with carfentanil decreased. During this period, the number of deaths with any fentanyl analog detected instead increased, mainly driven by acrylfentanyl (202 deaths) and furanylfentanyl (192 deaths). The number of deaths with carfentanil present in other states followed a similar pattern, with peaks occurring slightly after those in Ohio. During the first half of 2017, seven states reported detecting carfentanil in overdose deaths, compared with three during the second half of 2016; the number of counties in which overdose deaths with carfentanil present occurred increased from 54 to 77.

**FIGURE:**
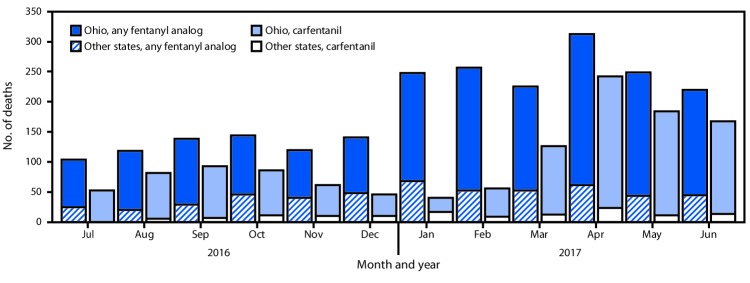
Number of overdose deaths with carfentanil and any fentanyl analog detected* — Ohio and nine other SUDORS states,^†^ July 2016–June 2017 **Abbreviation:** SUDORS = State Unintentional Drug Overdose Reporting System. * “Any fentanyl analog” includes carfentanil, so the categories are not mutually exclusive. **^†^** Kentucky, Maine, Massachusetts, New Hampshire, New Mexico, Oklahoma, Rhode Island, West Virginia, and Wisconsin.

In 2015, CDC issued a nationwide public health advisory about increases in fentanyl-related overdose deaths in multiple states (*5*), and in 2016 issued an update to that advisory to warn about increasing availability of fentanyl and fentanyl-related substances being pressed into counterfeit pills, and the potential for broad distribution across the United States (*6*). In response to findings in SUDORS data, on July 11, 2018, CDC issued a second update highlighting the emerging prevalence of fentanyl analogs contributing to opioid overdose deaths (*7*). Growing outbreaks associated with fentanyl analogs are occurring at a time when sharp increases in fentanyl overdose deaths are already straining the capacity of medical examiner and coroner offices and public health departments. The increasing array of fentanyl analogs highlights the need to build forensic toxicological testing capabilities to identify and report emerging threats and to enhance capacity to rapidly respond to evolving drug trends. The highly potent nature of many analogs, particularly carfentanil, might warrant multiple administrations of the effective opioid overdose reversal medication naloxone.

## Footnotes

* CDC’s Enhanced State Opioid Overdose Surveillance (ESOOS) program funded 12 states through a competitive application process in fiscal year 2016 and an additional 32 states and the District of Columbia in fiscal year 2017. States are funded to collect and share data on fatal and nonfatal opioid overdoses. The State Unintentional Drug Overdose Reporting System (SUDORS) is the component of ESOOS that collects data on fatal opioid overdoses. https://www.cdc.gov/drugoverdose/foa/state-opioid-mm.html.

† Data for the period from July 2016 through June 2017 were collected only by the 12 states that began reporting in August 2017 (Kentucky, Maine, Massachusetts, Missouri, New Hampshire, New Mexico, Ohio, Oklahoma, Pennsylvania, Rhode Island, West Virginia, and Wisconsin). At the time of reporting, data for Missouri and Pennsylvania were not complete and were therefore excluded.

§ SUDORS estimates of opioid-involved overdose deaths might differ from those of the National Vital Statistics System because SUDORS uses preliminary death certificate data and collects additional information from medical examiner and coroner reports, which are abstracted within 8 months of death. In SUDORS, an opioid-involved overdose death either was identified through review of the medical examiner/coroner report or had *International Classification of Disease, Tenth Revision* (ICD-10) underlying cause-of-death codes X40–44 (unintentional) or Y10–Y14 (undetermined) and multiple cause-of-death codes of T40.0, T40.1, T40.2, T40.3, T40.4, or T40.6 on the death certificate. Data for this report were downloaded on April 25, 2018, and might differ from reports using earlier data.

¶ Fentanyl analogs detected in at least one death: 3-methylfentanyl, 4-fluorobutyrfentanyl, 4-fluorofentanyl, 4-fluoroisobutyrfentanyl, acetylfentanyl, acrylfentanyl, butyrylfentanyl, carfentanil, cyclopropylfentanyl, cyclopentylfentanyl, furanylethylfentanyl, furanylfentanyl, isobutyrylfentanyl, and tetrahydrofuranylfentanyl. Decedents might have tested positive for more than one analog, as well as for other opioid and nonopioid substances. Multiple substances could have been used separately or mixed together, either with or without the decedents’ knowledge.
